# The Disease of Inebriety from Alcohol, Opium, and Other Narcotic Drugs

**Published:** 1894-11

**Authors:** 


The Disease of Inebriety from Alcohol, Opium and Other Narcotic Drugs. Its Etiology, Pathology, Treatment, and Medico-Legal Relations, arranged and compiled by the American Association for the Study and Cure of Inebriety. New York: E. B. Treat, Publisher, No. 5 Cooper Union. 1893. Price, $2.75.

The society at whose instance this book is published, and whose name is given above, was organized in 1870 in New York city. Its members were composed of physicians connected with asylums for inebriates, and others interested in the scientific study of the drink problem.

This association holds: First, that inebriety is a disease; second, it is curable, as are other diseases; third, the constitutional tendency to this disease may be inherited or acquired; fourth, that alcohol has its place in the arts and sciences, but as a medicine it is classed as a poison, and its internal use is always more or less dangerous; fifth, all methods of treatment hitherto employed have not recognized the disordered physical condition caused by alcohol, opium, or other narcotics, and hence have proved inadequate in its cure; hence, there is urgent need for special hospitals; sixth, the association urges that every large city should have its local and temporary hospital for both the reception and care of inebriates; seventh, it is the duty of the civil authorities to recognize inebriety as a disease, and to provide means for its scientific treatment in place of the usual penal methods of fines and imprisonment; eighth, the officers of such hospitals and asylums should have ample legal power over their patients.

The association finds "it is a curious fact that inebriety was recognized as a disease long before insanity was thought to be other than spiritual madness and a possession of the devil." For over a century the disease of insanity was denied and contested. Inebriety is passing the same ordeal of ignorant opposition and criticism, notwithstanding it has been recognized by a majority of the leading physicians of the world to-day.

The disease inebriety afflicts two great classes of patients—inebriates and dipsomaniacs.

Inebriates are divided into several sub-classes: Accidental inebriates, emotional inebriates, solitary inebriates, and pauper inebriates. The accidental cases are those that occur from the surroundings of the patients, the company they keep, and so on. The emotional inebriates belong to the class on the borders of hysteria with feeble will; all the time suffering from constitutional unrest and emotional struggles to attain the impossible. The solitary cases use alcoholic liquors secretly, and are morbidly sensitive about their infirmity. The pauper inebriates it is hardly necessary to describe.

The second great class, the dipsomaniacs are characterized by the suddenness with which the drink-habit comes on, its gradual dying out, and the occurrence of an interval of freedom. This is considered a true neurosis, and a branch of the family of epileptics, insane hysterias, and paranoic.

The dipsomaniacs occur in three forms: The acute, the periodic, and the chronic.

The inebriate diathesis is recognized as a constitutional proclivity or neurosis, which impels to the inordinate use of narcotics, including the hurtful use of opium, chloral, and cocaine, as well as of alcohol.

This tendency is classed as a mania and is called *narcomania*.

Chapters are devoted to the causes of inebriety; among which may be mentioñed traumatism and adversity.

Those who use alcohol in any form are more liable to sunstroke and apoplexia. Instances are cited of the occurrence of such cases on the public streets, where, on account of a smell of alcohol on the breath, the patient has been sent to the station-house instead of the hospital, and thus death supervened without any effort being made to avert such an event.

The relation of inebriety to consumption is also discussed, and the effects of beer and alcohol on the mental functions shown and its transmission by heredity investigated. Much more might be said, but enough is here given to show the scope of the book, its practical character, and its value to the physician. Written by physicians for physicians, there can be no question of its truthfulness and its freedom from sensational, unsupported statements, for partisan or missionary purposes.

It might be well to add that the binding and general appearance of the books are in uniformity with the other publications of E. B. Treat, so frequently noticed in these pages.

